# Associations Between Practice-Related Changes in Motor Performance and Muscle Activity in Healthy Individuals: A Systematic Review

**DOI:** 10.1186/s40798-018-0123-6

**Published:** 2018-02-08

**Authors:** Dennis Brueckner, Rainer Kiss, Thomas Muehlbauer

**Affiliations:** 1 Division of Sports Medicine and Engineering, Hochschule Koblenz—University of Applied Sciences, Remagen, Germany; 2 Division of Movement and Training Sciences/Biomechanics of Sport, University of Duisburg-Essen, Gladbecker Str. 182, 45141 Essen, Germany; 3 Department of Health and Social Affairs, FHM Bielefeld—University of Applied Science, Bielefeld, Germany

**Keywords:** Motor learning, Skill acquisition, Muscle activation, Electromyography (EMG), Muscles, Agonist, Antagonist, EMG amplitude and duration, Human

## Abstract

**Background:**

A well-learned motor skill is characterized by the efficient activation of muscles that are involved in movement execution. However, it is unclear if practice-related changes in motor performance correlate with those in quantitative markers of muscle activity and if so, whether the association is different with respect to the investigated muscle (i.e., agonist and antagonist) and quantitative myoelectric parameter.

Thus, we conducted a systematic review and characterized associations between practice-related changes in motor performance and muscle activity in healthy individuals.

**Methods:**

A computerized systematic literature search was performed in the electronic databases PubMed, Web of Science, and SPORTDiscus up to September 2017 to capture all relevant articles.

A systematic approach was applied to evaluate the 1670 articles identified for initial review. Studies were included only if they investigated healthy subjects aged 6 years and older and tested at least one measure of motor performance (e.g., error score, movement time) and quantitative muscle activity (i.e., amplitude domain: iEMG [integrated electromyography], RMS [root mean square]; time domain: duration of muscle activity, time to peak muscle activation). In total, 24 studies met the inclusionary criteria for review.

The included studies were coded for the following criteria: age, learning task, practice modality, and investigated muscles (i.e., agonist and antagonist). Correlation coefficients for the relationship of motor performance changes with changes in electromyography (EMG) amplitude, and duration were extracted, transformed (i.e., Fisher’s *z*-transformed *r*_z_ value), aggregated (i.e., weighted mean *r*_z_ value), and back-transformed to *r* values. To increase sample size, we additionally extracted pre and post practice data for motor performance and myoelectric variables and calculated percent change values as well as associations between both. Correlations were classified according to their magnitude (i.e., small *r* ≤ 0.69, medium *r* ≤ 0.89, large *r* ≥ 0.90).

**Results:**

Five studies reported correlation coefficients for the association between practice-related alterations in motor performance and EMG activity. We found small associations (range *r* = 0.015–0.50) of practice-related changes in motor performance with measures of agonist and antagonist EMG amplitude and duration. A secondary analysis (17 studies) that was based on the calculation of percent change values also revealed small correlations for changes in motor performance with agonist (*r* = − 0.25, 11 studies) and antagonist (*r* = − 0.24, 7 studies) EMG amplitude as well as agonist (*r* = 0.46, 8 studies) and antagonist (*r* = 0.29, 5 studies) EMG duration.

**Conclusions:**

Our systematic review showed small-sized correlations between practice-related changes in motor performance and agonist and antagonist EMG amplitude and duration in healthy individuals. These findings indicate that practice-related changes can only partly be explained by quantitative myoelectric measures. Thus, future studies investigating biomechanical mechanisms of practice-related changes in motor performance should additionally include qualitative measures of muscle activity (e.g., timing of muscle activity, level of coactivation) and other biomechanical variables (i.e., kinetics, kinematics).

**Electronic supplementary material:**

The online version of this article (10.1186/s40798-018-0123-6) contains supplementary material, which is available to authorized users.

## Key Points

- The present systematic review characterized associations between practice-related changes in motor performance and muscle activity in healthy individuals.
- Irrespective of the investigated myoelectric parameter (i.e., amplitude and duration) and muscle (i.e., agonist and antagonist), our analyses revealed small-sized correlations between changes in motor performance and muscle activity following motor practice.
- The observed small associations imply that practice-related changes in motor performance can only partly be explained by quantitative myoelectric measures, and thus, we recommend to additionally include qualitative measures of muscle activity (e.g., timing of muscle activity, level of coactivation) and other biomechanical variables (i.e., kinetics, kinematics) in future investigations.

## Background

Motor learning is defined as the changes, associated with practice or experience, in internal processes that determine a person’s capability for producing a motor skill [1]. For example, as learning a motor skill progresses, movement error and duration are reduced. These performance improvements are related to changes in the gradation and timing of force produced by the muscles involved in the skilled performance. The level and duration of muscle involvement during execution of a learned movement task is reflected in their myoelectric activity that can be recorded and displayed by the use of electromyography (EMG).

The effects of motor practice on myoelectric activity have been investigated for more than six decades. One of the first studies [2], conducted in the late 1950s, investigated relatively simple movements, i.e., participants practiced filing and chiseling for 2 to 6 weeks. For both tasks, practice resulted in an increased movement fluidity and a change in the EMG pattern from agonist-antagonist coactivation early in practice to reciprocal activation of both muscles late in practice. Ten years later, Kamon and Gormley [3] confirmed this result using a more complex movement task. In their experiment, subjects practiced the single knee circle mount on the horizontal bar. Over the 3-month practice period, they found a fluent execution of the exercise that was accompanied by a shift from continuous and overlapped muscle activity to phasic muscle activation. Thus, reciprocal, phasic, or sequential muscle activation seems to be qualitative indicators of resulting changes in the muscle activation pattern due to motor practice.

Further research tried to extend these findings with the aim to establish quantitative parameters of practice-related myoelectric changes. While some authors reported decreases [4, 5] in muscle activity following practice, others found increases [6, 7] or no changes [8, 9]. Therefore, the current evidence is conflicting, with a lack of studies systematically reviewing practice-related myoelectric changes associated with those in motor performance in healthy subjects. Moreover, previous studies primarily examined the effects of practice on measures of motor performance and muscle activity, separately, but did not report the relationship between the two.

Consequently, there is still a gap in our knowledge regarding potential associations between practice-induced adaptations in motor performance and muscle activity. Further, if there is such an association, information on the direction (i.e., positive or negative) and size (i.e., small, medium, or large) of the correlations need to be examined. A review of this topic will provide a better understanding of motor control processes and is suitable to provide information on how these processes change with skill acquisition. From a more practical point of view, the presented line of research is needed to determine the influence of motor practice on myoelectric activity in order to get insights that can be used for the design and evaluation of practice programs. Thus, the aim of this systematic literature review was to characterize associations between practice-related changes in motor performance and muscle activity in healthy individuals considering different well-established EMG variables (i.e., amplitude domain: integrated EMG [iEMG], root mean square [RMS]; time domain: duration of muscle activity, time to peak muscle activation). Since motor learning is characterized by a reduction in the force level and the time required to execute the practiced task, EMG amplitude and duration should also decrease, especially for the agonist muscle.

## Methods

### Literature Search

A computerized systematic literature search was conducted in PubMed, Web of Science, and SPORTDiscus from January 1950 up to September 2017. The following Boolean search strategy was applied using the operators AND, OR, NOT: (((motor learning OR motor practice OR skill acquisition) AND (muscle activity OR muscle activation OR EMG analysis OR electromyographic activity OR neuromuscular activity) NOT (patients OR disease))). The search was limited to full-text original articles, human species, and English language. Further, we checked the reference lists of each included article in an effort to identify additional suitable studies for inclusion in the database.

### Selection Criteria

To be eligible for inclusion, studies had to meet the following criteria: (a) participants of the experimental groups had to be healthy subjects and (b) at least one measure of motor performance and muscle activity had to be assessed in the study. Studies were excluded if (a) they investigated children (6–12 years), adolescents (13–18 years), seniors (≥ 65 years), patients, or people with diseases; (b) it was not possible to extract pre and post practice values from the results section; or (c) authors did not reply to our inquiries sent by email. Based on the predefined inclusion and exclusion criteria, two independent reviewers (DB, TM) screened potentially relevant papers by analyzing the titles, abstracts, and full texts of the respective articles to elucidate their eligibility. If no consensus was achieved between the two reviewers, a third reviewer (RK) was contacted.

### Coding of Studies

Each study was coded for the following variables: number of participants, age, learning task, practice modality, investigated muscle, motor performance, and muscle activity outcomes. For the latter one, we divided in measures of EMG amplitude (e.g., iEMG, RMS) and duration (e.g., duration of muscle activity, time to peak activation). For studies that reported multiple parameters within these outcome categories, the most representative parameter was included for further analysis. With regard to EMG amplitude, iEMG was defined as the most important variable. In terms of EMG duration, duration of muscle activity was used. As a function of the respective motor performance measure (i.e., error score or number of successful hits), the pre to post practice change can be negative or positive. Thus, a negative percent change value would indicate practice-related performance improvements (i.e., decrease in error score), and a positive percent change value would indicate a performance decrement (i.e., increase in error score) following practice.

#### Statistical Analyses

In a first approach, associations between practice-related changes in motor performance and muscle activity were assessed using the reported Pearson product-moment correlation coefficient (*r* value). To pool *r* values derived from different studies, “Fisher’s *z*′ transformation” was used, i.e., Pearson product-moment correlation coefficients were converted to the normally distributed variable *z*′ (i.e., *z*-transformed *r*_*z*_ value). The formula for the transformation is (Eq. 1):

$$ {z}^{\hbox{'}}=0.5\left[\ln \left(1+r\right)-\ln \left(1-r\right)\right] $$

where ln is the natural logarithm [10]. In addition, the included studies were weighted according to the magnitude of the respective standard error (*SE*). The formula for the calculation of the *SE* is (Eq. 2):

$$ SE=1/\surd \left(N-3\right) $$

where *N* stands for the respective sample size [10]. Afterwards, weighted mean *r*_*z*_ values were computed. To classify and interpret the correlation sizes, *r*_*z*_ values were back-transformed to *r* values. Based on the recommendations of Vincent [11], values of 0 ≤ *r* ≤ 0.69 indicate small, 0.70 ≤ *r* ≤ 0.89 medium, and *r* ≥ 0.90 large sizes of correlation. In a second approach, pre and post practice data for motor performance and myoelectric variables were extracted from other studies to calculate percent change values. Afterwards, associations between practice-related changes in motor performance and muscle activity were computed.

## Results

### Study Characteristics

**Fig. 1:**
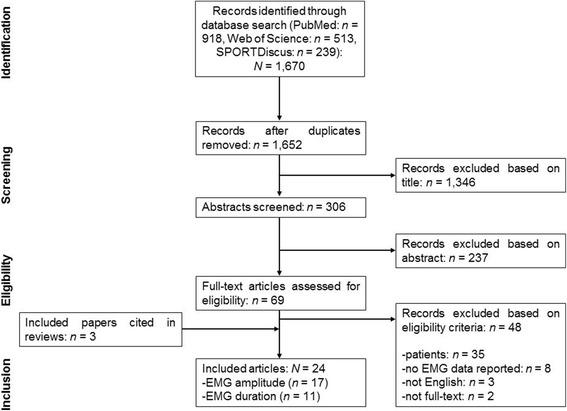
Flow chart describing the systematic literature search. *EMG* electromyography

Figure 1 displays a flow chart that illustrates the different stages of the systematic literature search and the selection of articles over the course of the search. The initial search identified 1670 studies that were potentially eligible for inclusion. After removal of duplicates and exclusion of ineligible articles, 21 studies remained. We identified another three articles from the reference lists of the included articles. Therefore, 24 studies were included in the final analysis with 17 and 11 studies that investigated parameters of EMG amplitude and duration, respectively.

Table 1 illustrates the main characteristics of the included studies (*n* = 17) examining practice-induced adaptations on motor performance and measures of EMG amplitude. Fourteen studies (*n* = 232 subjects) were performed with young adults only [4–9, 12–19], one study (*n* = 12) with young and middle-aged adults [20], one study (*n* = 28) with young and old adults [21], and one study (*n* = 22 subjects) did not report subjects’ age [22]. Maximal effort tasks (i.e., fast/ballistic accurate movements) were investigated in five studies [6, 16, 19, 20, 22] and submaximal effort tasks (i.e., target oriented or time/velocity constrained) were examined in 12 studies [4, 5, 7–9, 12–15, 17, 18, 21]. The literature search revealed a number of practice sessions ranging from one to ten. The number of trials per session ranged from ten to 200. One study reported a duration of 16 min per session [5]. The number of investigated agonist and antagonist muscles ranged from one to two.

**Table 1:** Studies (*n* = 17) examining learning effects on motor performance and measures of EMG amplitude

Study	Number of subjects; age (range or mean ± SD)	Learning task; practice modality	Investigated muscles
Hobart et al. [12]	31; 20–30 years	Submaximal effort task: target-oriented underhand ball toss; 1 session; 150 trials/session	Agonist muscles: pectoralis major, anterior deltoideus; Antagonist muscles: posterior deltoideus, triceps brachii
Payton et al. [14]	27; 20–34 years	Submaximal effort task: target-oriented propelling of a plastic disc; 1 session, 100 trials/session	Agonist muscle: abductor digiti quinti
Hobart et al. [13]	20; 20–30 years	Submaximal effort task: target-oriented underhand ball toss; 1 session; 104 trials/session	Agonist muscle anterior deltoideus; Antagonist muscle: posterior deltoideus
Moore and Marteniuk [4]	8; college-aged	Submaximal effort task: time-constrained (200 ms or 500 ms) 45° horizontal forearm extension; 4 sessions, 100 trials/session	Agonist muscle: triceps brachii; Antagonist muscle: biceps brachii
Darling and Cooke [22]	22; NR	Maximal effort task: target-oriented elbow flexion and extension (step tracking) with increased movement velocity; 1 session, 120 trials/session	Agonist and antagonist muscle: biceps brachii, triceps brachii
Engelhorn [9]	16; 21 years	Submaximal effort task: time-constrained horizontal elbow flexion; 2 sessions, 120 trials/session	Agonist muscle: biceps brachii; Antagonist muscle: triceps brachii
Dugas and Marteniuk [15]	16; college-aged	Submaximal effort task: target-oriented 70° and time-constrained (900 ms) forearm extension; 2 sessions, 100 trials/session	Agonist muscle: triceps brachii; Antagonist muscle: biceps brachii
Corcos et al. [19]	5; 20–25 years	Maximal effort task: rapid elbow flexion; 7 sessions, 200 trials/session	Agonist muscle: biceps brachii; Antagonist muscle: triceps brachii
Heise [7]	18; 22 ± 2 years	Submaximal effort task: target-oriented multi-joint throw; 1 session, 55 trials/session	Agonist muscles: triceps brachii, posterior deltoideus
Gabriel and Boucher [6]	18; 26 ± 3 years	Maximal effort task: target-orientated rapid (as fast as possible) elbow flexion (0° to 75°-90°); 4 sessions, 100 trials/session	Agonist muscle: biceps brachii; Antagonist muscle: triceps brachii
Aggelousis et al. [8]	41; 19–26 years	Submaximal effort task: target-oriented ball throw by performing an elbow flexion; 1 session, 90 trials/session	Agonist muscles: biceps brachii, brachioradialis; Antagonist muscles: triceps brachii, anconeus
Gabriel [16]	8; 25–30 years	Maximal effort task: target-orientated rapid (as fast as possible) elbow flexion; 4 sessions, 100 trials/session	Agonist muscle: biceps brachii; Antagonist muscle: triceps brachii
Lay et al. [5]	6; 18–21 years	Submaximal effort task: ergometer rowing at a fixed power output (100 W); 10 sessions, 16 min/session	Agonist muscles: vastus lateralis, biceps brachii
Christou et al. [21]	14; 24 ± 2 years 14; 72 ± 4 years (excluded from data analysis)	Submaximal effort task: target-oriented isometric contractions (abduction of the index finger); 1 session including 5 blocks, 20 trials/block	Agonist muscle: interosseus dorsalis I; Antagonist muscle: interosseus palmares II
Klein Breteler et al. [17]	9; 29 years	Submaximal effort task: manual spelling of words; 1 session including 7 blocks, 42 trials/block	Agonist and antagonist muscles: dorsal interosseus, abductor pollicis brevis, flexor pollicis brevis, abductor digiti minimi, flexor digitorum superficialis
Hasson et al. [18]	9; 25 ± 4 year	Submaximal effort task: target-oriented force production while maintaining a constant pedaling speed; 1 session, 18 trials/session	Agonist and antagonist muscles: tibialis anterior, soleus, vastus lateralis, medial gastrocnemius, rectus femoris, semitendinosus
Liang et al. [20]	12; 22–50 years	Maximal effort task: ballistic (maintain maximum velocity) wrist flexion; 10 sessions, 10 trials/session	Agonist muscle: flexor carpi radialis; Antagonist muscle: extensor carpi radialis

*EMG* electromyography, *NR* not reported, *SD* standard deviation

Table 2 shows the main characteristics of the included studies (*n* = 11) that examined practice-related changes on motor performance and measures of EMG duration. Nine studies (*n* = 159 subjects) were conducted in young adults only [6, 9, 13, 16, 23–27], one study (*n* = 12) with young and middle-aged adults [20], and one study (*n* = 28) with young and old adults [21]. Due to the possible influence of biological aging, data for the old adults were excluded from our data analyses. Maximal and submaximal effort tasks were used in four [6, 16, 20, 23] and seven studies [9, 13, 21, 24–27], respectively. One to ten practice sessions were performed that included ten to 120 trials per session. The number of studied agonist and antagonist muscles ranged from one to two.

**Table 2:** Studies (*n* = 11) examining learning effects on motor performance and measures of EMG duration

Study	Number of subjects; age (range or mean ± SD)	Learning task; practice modality	Investigated muscles
Hobart et al. [13]	20; 20–30 years	Submaximal effort task: target-oriented underhand ball toss; 1 session; 104 trials/session	Agonist muscle: anterior deltoideus; Antagonist muscle: posterior deltoideus
Ludwig [27]	12; 18–22 years	Submaximal effort task: target-oriented elbow extension; 1 sessions, 100 trials/session	Agonist muscle: triceps brachii; Antagonist muscles: biceps brachii
Normand et al. [23]	40; 19–35 years	Maximal effort task: target-oriented (maximum speed) horizontal arm adduction followed by a forearm flexion; 8 sessions, 100 trials/session	Agonist muscles: pectoralis major, biceps brachii Antagonist muscles: posterior deltoideus, triceps brachii
McGrain [24]	16; college-aged	Submaximal effort task: velocity-constrained (5 mph) knee extension; 1 session, 20 trials/session	Agonist muscles: vastus lateralis, vastus medialis
Engelhorn [9]	16; 21 years	Submaximal effort task: target-oriented horizontal elbow flexion; 2 sessions, 120 trials/session	Agonist muscle: biceps brachii; Antagonist muscle: triceps brachii
Gabriel and Boucher [25]	17; 19–32 years	Submaximal effort task: target-orientated elbow flexion (0° to 75°–90°); 4 sessions, 100 trials/session	Antagonist muscle: triceps brachii
Morrison and Anson [26]	12; 18–25 years	Submaximal effort task: target-orientated dart throwing task; 2 sessions, 80 trials/session	Agonist muscle: triceps brachii; Antagonist muscles: brachioradialis, biceps brachii
Gabriel and Boucher [6]	18; 26 ± 3 years	Maximal effort task: target-orientated rapid (as fast as possible) elbow flexion (0° to 75°–90°); 4 sessions, 100 trials/session	Agonist muscle: biceps brachii; Antagonist muscle: triceps brachii
Gabriel [16]	8; 25–30 years	Maximal effort task: target-orientated rapid (as fast as possible) elbow flexion; 4 sessions, 100 trials/session	Agonist muscle: biceps brachii; Antagonist muscle: triceps brachii
Christou et al. [21]	14; 24 ± 2 years 14; 72 ± 4 years (excluded from data analysis)	Submaximal effort task: target-oriented isometric contractions (abduction of the index finger); 1 session including 5 blocks, 20 trials/block	Agonist muscle: interosseus dorsalis I; Antagonist muscle: interosseus palmares II
Liang et al. [20]	12; 22–50 years	Maximal effort task: ballistic (maintain maximum velocity) wrist flexion; 10 sessions, 10 trials/session	Agonist muscle: flexor carpi radialis; Antagonist muscle: extensor carpi radialis

*EMG* electromyography, *SD* standard deviation

### Associations Between Practice-related Changes in Motor Performance and Muscle Activity

**Fig. 2:**
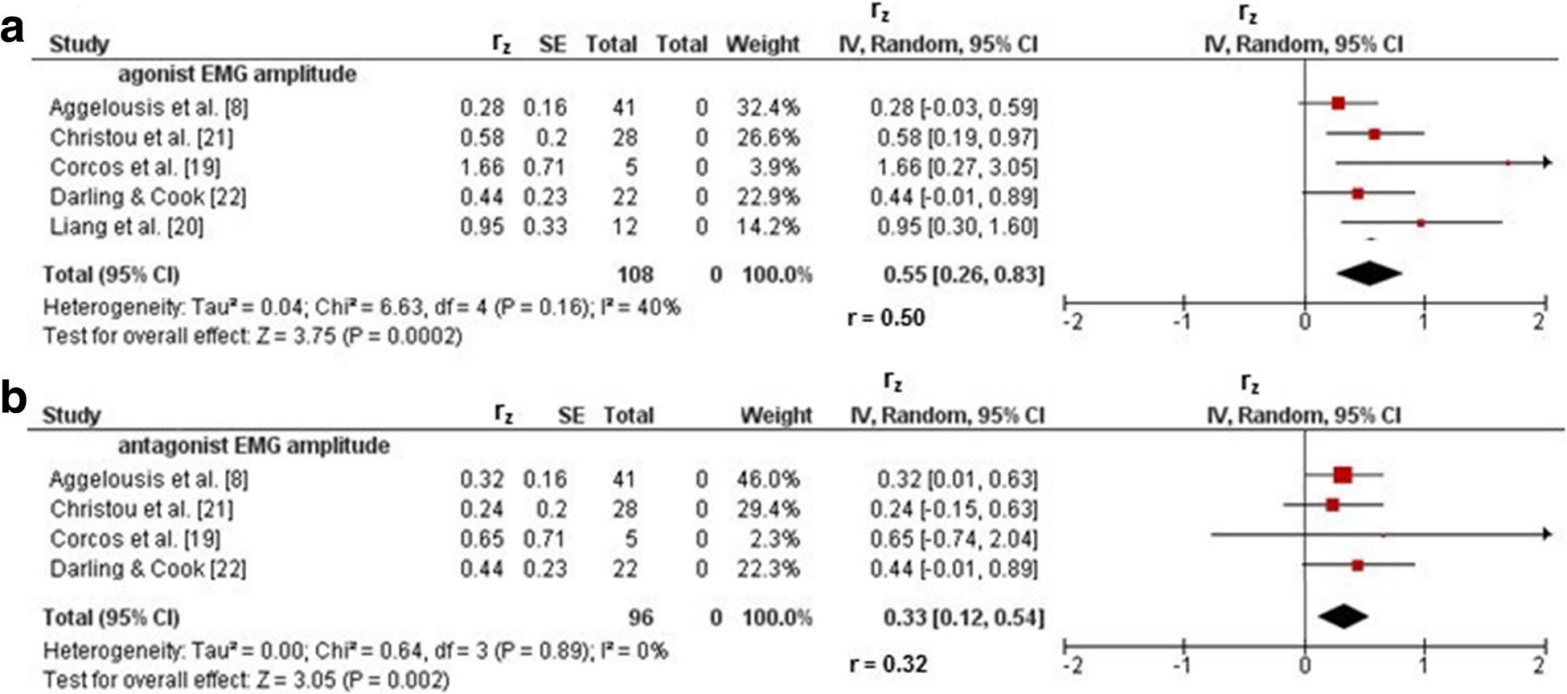
Associations between practice-related changes in motor performance and EMG amplitude in healthy subjects. **a** Correlation coefficients for the agonist EMG amplitude. **b** Correlation coefficients for the antagonist EMG amplitude. *CI* confidence interval, *df* degrees of freedom, *EMG* electromyography, *IV* inverse variance, *r* back-transformed Pearson’s correlation coefficients, *r*_*z*_ weighted *z*-transformed Pearson’s correlation coefficients, *SE* standard error

Five studies [8, 19–22] reported correlation coefficients for the association between practice-related changes in motor performance and myoelectric activity. Figure 2a, b illustrates the relationships of motor performance with agonist and antagonist EMG amplitude, respectively. Weighted mean *r*_z_ values amounted to 0.55 for measures of agonist EMG amplitude (*I*^2^ = 40%, chi-square = 6.63, *df* = 4, *p* = 0.16, five studies: [8, 19–22]) and 0.33 for outcomes of antagonist EMG amplitude (*I*^2^ = 0%, chi-square = 0.64, *df* = 3, *p* = 0.002, four studies: [8, 19, 21, 22]). Back-transformed *r* values of 0.50 (*R*^2^ = 25%) and 0.32 (*R*^2^ = 10%) indicated small correlations. Additionally, one study [21] reported a small correlation of motor performance with measures of agonist (*r*_z_ = 0.36, *r* = 0.347, *R*^2^ = 12%) and antagonist (*r*_z_ = 0.02, *r* = 0.015, *R*^2^ = 0%) EMG duration.

**Fig. 3:**
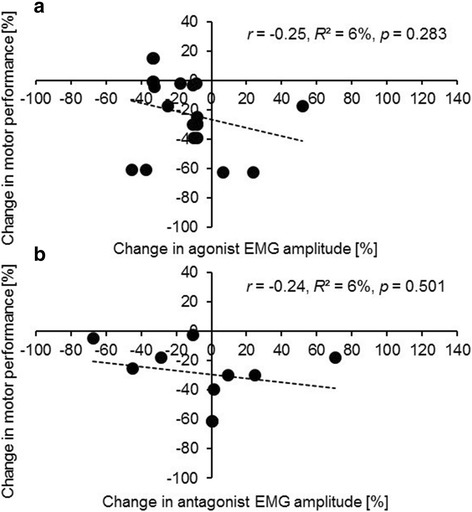
Associations between practice-related changes in motor performance and EMG amplitude in healthy subjects extracted from 11 studies. **a** Data points for the agonist EMG amplitude (11 studies, 20 data points). **b** Data points for the antagonist EMG amplitude (7 studies, 10 data points). The number of data points is larger compared to the number of studies because a few studies studied multiple muscles. Muscles acting as both agonist and antagonist were excluded from data analyses. *EMG* electromyography, *r* Pearson’s correlation coefficients, *R*^*2*^ coefficient of determination

**Fig. 4:**
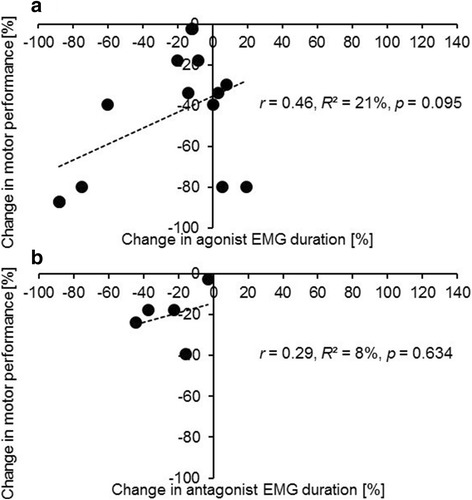
Associations between practice-related changes in motor performance and EMG duration in healthy subjects extracted from 9 studies. **a** Data points for the agonist EMG duration (8 studies, 14 data points). **b** Data points for the antagonist EMG duration (5 studies, 5 data points). The number of data points is larger compared to the number of studies because a few studies studied multiple muscles. Muscles acting as both agonist and antagonist were excluded from data analyses. *EMG* electromyography, *r* Pearson’s correlation coefficients, *R*^*2*^ coefficient of determination

In addition, pre and post practice data for motor performance and myoelectric activity were extracted from 17 studies to calculate percent change values and associations between both. Figure 3 displays scatterplots for changes in motor performance with agonist (Fig. 3a) and antagonist EMG amplitude (Fig. 3b). Our analysis revealed negative, small associations of motor performance with agonist (*r* = − 0.25, *R*^2^ = 6%, *p* = 0.283, 11 studies, 20 data points; Fig. 3a) and antagonist (*r* = − 0.24, *R*^2^ = 6%, *p* = 0.043, 7 studies, 10 data points; Fig. 3b) EMG amplitude (Additional file 1: Table S1). Figure 4 shows scatterplots for alterations in motor performance with EMG duration for agonist (Fig. 4a) and antagonist muscles (Fig. 4b). Our analysis yielded positive, small associations of motor performance with agonist (*r* = 0.46, *R*^2^ = 21%, *p* = 0.095, 8 studies, 14 data points; Fig. 4a) and antagonist (*r* = 0.29, *R*^2^ = 8%, *p* = 0.634, 5 studies, 5 data points; Fig. 4b) EMG duration (Additional file 2: Table S2).

## Discussion

The present systematic literature review characterized associations between practice-related changes in motor performance and muscle activity in healthy individuals considering different myoelectric variables (i.e., agonist and antagonist EMG amplitude and duration). This research is important for a better understanding of myoelectric adaptation processes initiated by motor practice. Especially, we focused our analyses on well-established EMG measures in the amplitude (e.g., iEMG, RMS) and time (e.g., time to peak muscle activation) domain because these are particularly related to modifications in the spatial and temporal characteristics of muscle force production following practice. We hypothesized that improvements in motor performance following practice are accompanied by reductions in EMG amplitude and duration, especially observed in the agonist muscle. We found only small-sized associations between practice-related alterations in motor performance and agonist and antagonist muscle activity. This finding was independent from the used data source, that is, associations reported in the literature and correlation coefficients additionally calculated from extracted pre and post practice data for measures of motor performance and myoelectric activity. In general, our findings indicate that practice-related changes in motor performance might only partly be explained by changes in quantitative myoelectric parameters (i.e., agonist and antagonist EMG amplitude and duration). A possible reason for the small-sized associations between practice-related alterations in motor performance and agonist and antagonist muscle activity could be the concurrent effect of skill acquisition on qualitative EMG parameters, such as the timing of muscle activity or the amount of coactivation. In this regard, previous research [2–4] showed that during motor practice, the EMG pattern alters from continuous and overlapped muscle activity (i.e., early in practice) to phasic and reciprocal activation (i.e., late in practice). As a consequence, further research is needed to determine the relationship between changes in motor performance and qualitative EMG variables due to motor practice. Additionally, besides the mentioned changes in qualitative myoelectric parameters, adaptations in kinetic (e.g., joint torques, forces) and kinematic (e.g., joint positions, angles, angular velocity, acceleration) variables might have influenced our findings of small-sized correlations between changes in motor performance and muscle activity following practice. For example, Corcos et al. [19] reported significant improvements in movement kinematics (e.g., increased peak moment velocity and acceleration) as a function of practice in young adults aged 20 to 25 years. Further, Christou et al. [21] showed that practicing an endpoint accuracy task resulted in a significantly reduced variability of the force trajectory, peak force, and time-to-peak force in healthy adults and was significantly related (range *r* = 0.394–0.585) to the improvement in time endpoint error. Thus, we recommend that future studies should add kinetic and kinematic analyses to the investigation of modifications due to motor practice.

The observed correlations differed in terms of their direction. More specifically, in four out of five studies that previously reported correlation coefficients between practice-related changes in motor performance and agonist EMG amplitude, the *r* value was positive. Contrary, a negative *r* value was obtained from the extracted pre and post practice data between motor performance and agonist EMG amplitude. However, the reason for this findings cannot unambiguously be explained using our data set because only three of the included 20 data points indicated an increase in myoelectric activity although motor performance improved as a result of practice. Additionally, positive associations were detected for practice-related changes in motor performance with agonist and antagonist EMG duration but negative relationships were found for motor performance with agonist and antagonist EMG amplitude. In other words, as motor performance improved agonist and antagonist EMG duration decreased, yet agonist and antagonist EMG amplitude increased. What are likely explanations for the observed difference in correlation direction? A decrease in agonist and antagonist EMG duration corresponds with reports [6, 16, 25] of reductions in movement time required to execute a practiced task. With regard to agonist EMG amplitude, an increase could be caused by the specific demands of the utilized practice task. More specifically, when the goal is to increase the speed of limb movement during practice (e.g., to perform fast accurate movements [19, 22]), this would lead to an increase in the magnitude of muscle activity (e.g., agonist EMG amplitude) as proposed by the “speed-sensitive strategy” [28]. In terms of antagonist EMG amplitude, an increment might be elicited by the specific role of antagonist muscles when performing the practice task. Contrary to agonist muscles that are responsible for efficient movement execution, antagonist muscles mainly have assistive or stabilizing function necessary to decelerate the limb movement that leads to extended myoelectric activation [12].

### Limitations

A limitation of this systematic review is that only five studies were found that reported correlation coefficients for alterations in motor performance associated with those in muscle activity. To increase sample size, we additionally extracted pre and post practice data for measures of motor performance and muscle activity from another 17 studies. From this, percent change values were calculated followed by correlational analyses. As a consequence, an indirect comparison of practice-related changes in motor performance with muscle activity was performed that requires substantiation by further studies that directly compare practice-related alterations in motor performance associated with changes in muscle activity via correlational analysis. Further, practice-related alterations in motor performance were assessed along with associated changes in quantitative (i.e., EMG amplitude, duration) but not in qualitative (i.e., timing of muscle activity, amount of coactivation) myoelectric variables or other biomechanical variables (i.e., kinetics and kinematics). Thus, we recommend that future studies should conduct comprehensive electromyographic examinations together with kinetic and kinematic analyses of pre and post practice data. In addition, the observed practice-related changes in muscle activity could be influenced by differences in the used practice task (i.e., maximal versus submaximal effort tasks). Maximal effort tasks such as fast/ballistic accurate movements were investigated in six studies and require an increase in limb speed, and thus, an increase in EMG amplitude would be an appropriate change. Submaximal effort tasks were examined in 18 studies and include target-oriented and time-/velocity-constrained movements where the goal is to reduce movement error, time, or velocity. In this case, a decrease in EMG amplitude and duration would be an adequate adaptation. As a consequence, further research is needed to determine whether practice task category (i.e., maximal versus submaximal effort task) influences the relationship between practice-related changes in motor performance and muscle activity in healthy individuals. Finally, correlations do not show cause and effect. Hence, the investigated associations could be affected by other not yet examined variables such as alterations in qualitative (i.e., timing of muscle activity, amount of coactivation) myoelectric parameters or other biomechanical variables (i.e., kinetics, kinematics).

## Conclusions

The present systematic review revealed small-sized correlations between practice-related changes in motor performance and agonist and antagonist EMG amplitude and duration in healthy individuals. Our findings indicate that practice-related adaptations in motor performance of healthy persons can only partly be explained by changes in quantitative myoelectric measures. Consequently, future studies investigating practice-induced adaptations are advised to integrate qualitative myoelectric as well as other biomechanical variables (i.e., kinetics, kinematics).

## Additional files

Additional file 1: **Table S1.** Pre and post practice data for motor performance and EMG amplitude. (XLSX 37 kb)

Additional file 2: **Table S2.** Pre and post practice data for motor performance and EMG duration. (XLSX 31 kb)
